# The Relationship between Kinesiophobia, Emotional State, Functional State and Chronic Pain in Subjects with/without Temporomandibular Disorders

**DOI:** 10.3390/jcm13030848

**Published:** 2024-02-01

**Authors:** Tomasz Marciniak, Weronika Kruk-Majtyka, Patrycja Bobowik, Sławomir Marszałek

**Affiliations:** 1 Faculty of Rehabilitation, Józef Piłsudski Academy of Physical Education in Warsaw, 00-968 Warsaw, Poland; weronika.kruk@awf.edu.pl (W.K.-M.); patrycja.bobowik@awf.edu.pl (P.B.); 2 Department of Physiotherapy, Poznan University of Medical Sciences, 61-701 Poznan, Poland; 3 Faculty of Physical Education in Gorzow Wielkopolski, Poznan University of Physical Education, 61-871 Poznan, Poland

**Keywords:** kinesiophobia, temporomandibular disorders, TMD, functional limitations

## Abstract

Although there is growing evidence that kinesiophobia is correlated with temporomandibular disorders (TMD), its relationship with other characteristic TMD comorbidities, such as depression, anxiety, functional limitations, and pain in the TMD population, has rarely been investigated. This study aims to evaluate the relationship between kinesiophobia, emotional state, functional state and chronic pain in subjects both with and without TMD. A total of 94 subjects participated in the study and were divided into two groups (47 subjects each)—TMD (subjects with temporomandibular disorders) and nTMD (asymptomatic controls)—on the basis of the RDC/TMD protocol. All measurements were taken with self-administered questionnaires: TSK-TMD for kinesiophobia, PHQ-9 and GAD-7 for psychoemotional state, JFLS-20 for jaw functional limitations, and GCPS for chronic pain. The prevalence of kinesiophobia in the TMD group was 38.3% for moderate risk, and 61.7% for high risk. The TMD group showed significantly higher scores in all categories (kinesiophobia, depression, jaw functional limitations and chronic pain), with the exception of anxiety which was right at the cut-off point. Moreover, a significant correlation was found between kinesiophobia (TSK-TMD) and jaw functional limitations (JFLS-20). Results of this study could provide new insight into the relationship between kinesiophobia and TMD, further improving the diagnosis process.

## 1. Introduction

“Temporomandibular disorders” (TMD) is an umbrella term that refers to one of the main causes of orofacial pain, which consist of a variety of symptoms: myofascial pain or myalgia, intraarticular disorders (disc displacement with or without reduction) and temporomandibular joint pain (arthralgia) [1]. Previous studies showed the prevalence of TMD is between 3–12% in the general population [2,3,4], and indicated its etiology is multifactorial and includes a wide range of physical and psychoemotional factors, such as emotional stress, depression, anxiety, hormonal changes, occlusion etc. [5,6].

In responding to the emotional influence of TMD in recent years, some authors, in citing its recognition as an important component of chronic pain [7,8], claimed that kinesiophobia could also play a role in a broad spectrum of disorders in TMD.

Kinesiophobia, which is defined as the fear of movement, describes a situation where a patient has an excessive, irrational, and debilitating fear of physical movement and activity, which results from a feeling of vulnerability to painful injury or reinjury [9]. In TMD subjects the fear of movement of the jaw may affect some essential primary activities, such as eating, chewing, emotional expression, or communication [10]. Several studies assessed kinesiophobia and catastrophizing levels in TMD subjects by using standardized tools such as Tampa scale for kinesiophobia for temporomandibular disorders (TSK-TMD) [10,11,12], with some showing a stronger correlation with painful TMD, compared to nonpainful and control groups. On the other hand, other researchers found this relationship existed, regardless of the existence of pain [10]. It is worth mentioning that the prevalence of kinesiophobia in TMD has only been estimated in one previous study [11]; all other papers investigated the differences between study and control groups, but without showing actual prevalence.

In 2020, von Piekartz et al. [13] published official guidelines related to the international consensus on the most useful assessment tools that physical therapists used to evaluate patients with temporomandibular disorders and, in addition to other tools, recommended PHQ, GAD, JFLS, GCPS, and TSK-TMD questionnaires. Despite the publication of guidelines, no papers have been, to date, been published that investigate the relationship between kinesiophobia, emotional state, functional state and pain, and produce results that can be compared to each other in one global project. While individual studies have tried to address this question in different configurations, their use of different tools produced a limited and selective analysis [10,14,15,16,17].

From a clinical perspective, it seems to be important to establish this relationship because it may influence decisions about the therapy modalities that are in the patient’s best interest. The presence of kinesiophobia may indicate a need for treatment to be extended beyond just manual therapy.

In taking these different points into consideration, this study sought to (1) evaluate the relationship between kinesiophobia and psychoemotional state, functional state and chronic pain in subjects with temporomandibular disorders and healthy controls; (2) establish the prevalence of kinesiophobia in the studied population.

## 2. Materials and Methods

### 2.1. Material

The study involved 94 subjects (aged: 27.14 ± 6.19) aged between 18–45 years-of-age, which were included after providing their (written) consent to participate in the study of temporomandibular joint disorders (orofacial pain, intraarticular disorders, masticatory muscle pain) and associated symptoms. The study excluded those with rheumatoid diseases (e.g., rheumatoid arthritis (RA) and ankylosing spondylitis (AS)), whose jaw had previously been injured (e.g., bone fractures), whose temporomandibular joint ad been injured (e.g., contusions, dislocations), who had undergone facial surgical procedures (e.g., orthognathic surgery, TMJ surgery), or who withheld their written consent and/or did not fall into the study age range. 

All participants were divided into two groups based on the presence (TMD group) or absence (nTMD group, i.e., control group) of temporomandibular disorders symptoms, and this division was made on the basis of examination protocol taken from the research diagnostic criteria for temporomandibular disorders (RDC/TMD). After unsuitable candidates were excluded, the TMD group consisted of 47 subjects (10 males, 37 females) aged 27.5 ± 5.6, while the nTMD group consisted of the same number of participants (15 males, 32 females), who were aged 26.8 ± 6.8. 

The subjects in the nTMD group did not present any TMD signs nor symptoms, while the prevalence of different groups of symptoms in the TMD group was as follows: muscle pain (Group I) 60% (28 subjects); intraarticular disorders (Group II) 100% (47 subjects); temporomandibular joint pain 27.7% (13 subjects). The concurrence of two or more symptoms, which is very common in TMD patients [18,19,20,21], was also observed. The local ethical commission approved the study (signature SKE01-30/2021), after revisions were made to ensure the standards of the Helsinki Declaration, including all participants first providing written consent, were met. 

### 2.2. Methods

After the RDC/TMD examination protocol was completed, all subjects were asked to fill out questionnaires: the Tampa scale for kinesiophobia for temporomandibular disorders (TSK-TMD), patient health questionnaire (PHQ-9), generalized anxiety disorder (GAD-7), jaw functional limitation scale (JFLS-20) and graded chronic pain scale (GCSP). All of questionnaires are standardized and widely used in both clinical and research setups, and show good diagnostic value when applied to the assessment of the TMD population [8,13,16,22]. The whole RDC/TMD examination protocol was performed by a TMD specialist with more than a decade of experience.

TSK-TMD is an 18-item scale that measures levels of kinesiophobia (fear of movement) in subjects with temporomandibular disorder symptoms. Likert scale 1–4 is used to determine the intensity of the situation in each question: 1—strongly disagree, 2—somewhat disagree, 3—somewhat agree, 4—strongly agree. The total score is determined by summing the items: the lowest possible minimum score was 18, and the highest possible maximum was 72. The clinical classification of kinesiophobia was based on TSK-17 questionnaire cut-off points proposed by other researchers, and assessed as low risk (<17 points), moderate risk (17–37 points), and high risk (>37 points) [14,23,24].

Both PHQ-9 and GAD-7 questionnaires seek to assess the psychoemotional state of the subject by establishing the level of depression and anxiety, respectively. PHQ-9 consists of nine questions, which are each scored from 0 to 3 (possible maximum of 27 points in total) and GAD-7 consists of seven questions with the same scoring rules (possible maximum of 21 points). The cut-off point for both clinically significant depression and generalized anxiety disorder is set at 10 points, and the time perspective of both questionnaires is set at the last 14 days [25,26].

The next evaluative instrument used was JFLS-20, which seeks to identify any functional limitations from the last 30 days. Subjects were asked to indicate the severity of limitations of different activities, such as chewing different foods, swallowing, yawning, mouth opening, etc., on a scale from 0 to 10, with 0 meaning “no limitation” and 10 “severe limitation”. The global score is calculated via a algorithm proposed by [27].

The graded chronic pain scale (GCSP), which aims to assess pain levels from the last six months, was the last questionnaire to be implemented. In this study, only the results from the characteristic pain intensity (CPI) category will be used for further analysis [28].

### 2.3. Statistical Analysis

Data was analyzed using STATISTICA 13.0 (StatSoft). The Shapiro-Wilk test assessed the distribution of variables and Levene’s test determined homogeneity of variance. Both the *T*-test and Mann-Whitney U test were used. Effect sizes were assessed by applying Glass’s rank-biserial correlation coefficient. Cohen observes the effect size is low if the value of r varies around 0.1, medium if r varies around 0.3, and large if r varies over 0.5 [29]. An ANOVA for factorial designs was conducted, and Spearman’s rank correlation test was also performed, with a significance level set at *p* ≤ 0.05.

## 3. Results

The level of kinesiophobia in all subjects from both groups was assessed, showing there was nobody with a low risk of fear of movement. The results of the prevalence of kinesiophobia in subgroups, taken from the TSK-17 cut-off points put forward by Lira et al., Wertli et al. and Dupuis et al. [14,23,24], are presented in Table 1.

The analysis showed higher values in the TMD group. Significant differences were observed in TSK-TMD, PHQ-9 and GCPS (CPI), and also in JFLS-20 levels between groups (*p* < 0.001). The detailed results of this analysis are presented in Table 2.

**Figure 1:**
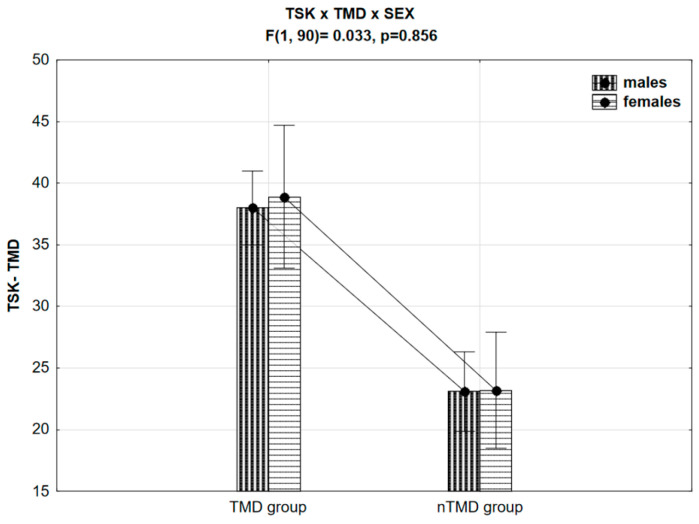
TSK-TMD differences, divided by gender and TMD presence.

In addition, no interactions between TSK-TMD × TMD × SEX (F(1, 90) = 0.033, *p* = 0.856, ƞ = 0.0003) were found. TSK-TMD levels in females were higher, compared to males, but not at a statistically significant level (F(1, 90) = 0.053, *p* = 0.818, ƞ = 0.0006). The results showed that kinesiophobia levels were significantly different between TMD and nTMD groups (F(1, 90) = 49.26, *p* < 0.01, ƞ = 0.354). The results of the analysis are presented in Figure 1.

Moreover, the TSK-TMD results were correlated with the functional limitations of participants (*p* < 0.001, r = 0.621), but not with emotional state nor chronic pain intensity. All results are shown in Table 3.

## 4. Discussion

From a clinical perspective, there is a strong need to screen for kinesiophobia levels in both TMD and nTMD subjects. It seems that the fear of movement is directly correlated with pain intensity, as well as the patient’s emotional state. This study therefore aimed to assess the kinesiophobia—psychoemotional state—functional state—pain relationship in subjects with temporomandibular disorders and healthy controls. The second study aim was to assess kinesiophobia prevalance in the studied TMD group.

The results showed that subjects with temporomandibular disorders (TMD group) had significantly higher levels of kinesiophobia (Z = 6.03, *p* < 0.01, R = 0.723), depression (Z = 3.78, *p* < 0.01, R = 0.453), chronic pain (Z = 5.13, *p* < 0.01, R = 0.615), and more severe functional limitations (Z = 6.16, *p* < 0.01, R = 0.737), compared to the asymptomatic group. Moreover, while TMD patients showed higher levels of anxiety (Z = 1.96, *p* = 0.05, R = 0.235), the difference with the control group was borderline statistically significant.

The lack of interaction between TSK-TMD × TMD × SEX means that although women show a higher levels of anxiety (compared to men), this difference is not statistically significant. On this basis, it can be assumed that the TSK-TMD questionnaire can be used in both women and men, without fear that one gender has a greater predisposition to kinesiophobia. On the other hand, women experience TMD symptoms more often, and also seek professional medical help more frequently (than men) [18,30,31]: both findings suggest researchers should refrain from claiming definite conclusions, and highlight the need for further research.

### 4.1. Kinesiophobia and Emotional Status

Although the connection between kinesiophobia and psychoemotional state has been investigated before, only a few studies used TMD-dedicated tools (e.g., TSK-TMD and PHQ questionnaires) [8,10] to assess kinesiophobia and depression levels (i.e., TSK-TMD and PHQ questionnaires) [8,10], finding, in both cases, significant differences between groups (painful TMD, nonpainful TMD, and control group). Other researchers who also assessed emotional status and kinesiophobia in TMD patients by using different measurement tools, such as the hospital anxiety and depression scale (HADS), reached similar conclusions [14]. The results of the current study are consistent with those obtained from previous studies, with the exception of the finding there is no correlation between kinesiophobia and depression levels.

Another aspect of the analysis that should be emphasized is the fear of movement in subjects with joint sounds (clicks due to disk displacement with reduction). Previous studies produced contradictory results on this, but mostly supported this correlation [10,32,33]. Poluha et al., analyzed this extensively, and concluded that subjects with joint sound (regardless of pain existence) showed higher kinesiophobia levels. The authors cited these potential explanations for this phenomenon by using the following arguments put forward by other researchers. First, it seems that the fear of movement is strongly related to mechanical jaw problems, such as sounds or locking [9,34]; second, the pain sensation of the click moment also influences this movement avoidance [35,36]; and finally, the movement-evoked pain conditions are commonly linked to fear of movement [37]. The click sound creates a suspicion the joint is malfunctioning, even in patients without pain [38,39]. Another hypothetical explanation for this could be that joint sounds create a feeling of embarrassment in patients, who therefore avoid movement, as stated by [33]. On the other hand, only Lira et al. displayed no correlation between temporomandibular joint sounds and kinesiophobia in TMD patients [14], but did not identify any possible causes.

The results of the current study are in accordance with the majority of the results available in the literature, as all of the participants experienced joint sounds, and simultaneously showed higher kinesiophobia levels than controls.

The latest systematic review by Dupuis et al. [24] concluded there is a need for further research that compares fear of movement to other similar constructs related to fear/anxiety associated with pain (e.g., pain catastrophizing scale), and claimed this would settle the validity of the TSK-TMD questionnaire. The current study tried to meet this demand by inspecting the kinesiophobia—depression —anxiety —chronic pain relationship, but failed to show a correlation. Moreover, anxiety levels between groups did not even reach the level of significance, and were just above the cut-off point. This could be due to a number of reasons, including the small sample size, the use of different diagnostic tools across individual articles (for example, HADS in the case of depression), the lack of catastrophizing assessment, and the correlation between emotional status and pain presented by others, including [40]. The general consensus holds there is a connection between depression, anxiety and pain perception. However, as the results of the current study did not show significant anxiety level differences between groups, which could potentially influence the emotional status—pain relationship indirectly, resulting in a lack of correlation between kinesiophobia, depression, anxiety and pain in the studied group.

### 4.2. Kinesiophobia and Functional Limitations (JFLS)

Current results showed a significant correlation between TSK-TMD and the results of JFLS-20, indicating that the functional limitations of TMD subjects increase with the level of kinesiophobia. This is consistent with Kim et al., who suggest that fear of movement may play an important role in mastication impairment [15], and even propose an innovation based on the premise that it would be very useful to compare subcategories from each questionnaire, meaning that jaw function, mastication, and communication for JFLS-20 would be compared with the kinesiophobia (low, moderate and high) risk expressed in TSK-TMD. This is a novelty that future research should consider, and possibly implement.

Some authors conclude that patient-reported outcomes, such as pain perception, pain catastrophizing, and kinesiophobia, are risk factors that seem to have more influence on jaw function than psychological distress, such as depression and anxiety. Current research also supports this by showing a significant correlation between TSK-TMD and JFLS, but not with PHQ or GAD scores. This is another indication that the PCS questionnaire should be used to explore the influence of catastrophizing on functional limitations.

Moreover, the cited study analyzed 145 subjects, more than the current study (94), which could be an influencing factor.

### 4.3. Kinesiophobia and Chronic Pain (GCPS)

Pain perception is subjective and can be assessed in two ways, first after mechanical induction by using an algometer; and second, with self-administered questionnaires (BPI or GCPS). To date, no studies have investigated the kinesiophobia—pain relationship in the TMD population by applying the Graded Chronic Pain Scale (GCPS). The results of this current study showed no correlation between TSK-TMD and GCPS scores, in contrast to previous studies that demonstrated kinesiophobia is a predictor of pain and disability in TMD patients, and therefore has an important clinical significance in the assessment and treatment of TMD [41]. The current study’s lack of significance could be due to the results from the characteristic pain intensity (CPI), rather than the global score of the questionnaire, being used for statistical analysis. Future studies should perhaps use the global score, and may well find it to be more informative.

### 4.4. Prevalence of Kinesiophobia in TMD

The prevalence of kinesiophobia in both groups (TMD and nTMD) was calculated and presented, and the results showed that nobody in either group presented a low risk of kinesiophobia. Most of the subjects in the TMD group presented a high risk of fear of movement and the majority of the nTMD group presented moderate risk. Scores in the TMD group were, in comparison to the controls, found to be significant in comparison with the controls. One flaw of the kinesiophobia classification, which should be emphasized, resulted in the cut-off points being taken directly from the TSK-17 classification with 17 questions, one less than the TSK-TMD. The risk levels were set at less than 17, 17–37 and more than 37 points for low- moderate- and high-risk, respectively. The TSK-17 scale was used because there are no TSK-TMD cut-off points in the literature.

This lack is both a limitation of this study and a signpost for future studies, as the creation of subscales (based on cut-off points dedicated to TSK-TMD) that determine levels of kinesiophobia would help to establish a risk assessment that could be used to classify patients, and better address their treatment plans.

To the best of our knowledge, only one paper has, to date, showed prevalence of kinesiophobia in the TMD population [11], in contrast to all the other studies cited in this paper, which merely compare study and control groups. The study group of this one paper only contained 28 subjects, whereas this study had 94, a figure that, while higher, still seems low when generalization of the results to the entire population is considered. Given this, future studies should consider larger study samples. 

### 4.5. Summary

The diagnostics of kinesiophobia in temporomandibular disorder patients seems to be crucial for future treatment selection, including the choice between manual therapy (or physical therapy in general) and other modalities, such as psychotherapy, behavioral therapy or drug administration.

Because the cause of the temporomandibular disorders is multifactorial, the approach to this type of patient should be global and multidisciplinary. In addition to considering the history of the TMJ and undertaking a clinical examination of it, an assessment of the levels of kinesiophobia should also be undertaken, as this seems to be one of the most important elements that affects patient performance. The identification of the risk factor could be a potential indicator that is used for prognostic reasons in assessments of pain chronicity.

There are ongoing scientific projects that aim to lowering kinesiophobia levels by searching for the most effective pain management and functional improvement tools that can be offered on top of conventional TMD treatment. This is important to remember, given that most of the studies cited in this paper only focused on depression and/or anxiety treatment [42].

In conclusion, when kinesiophobia is involved, other specialists should be involved in the multidisciplinary treatment team, as this will ultimately be in the best interests of temporomandibular disorder patients.

### 4.6. Study Limitations

The findings of this study should be interpreted in light of its limitations, of which the most important are the small sample size and the absence of the PCS questionnaire assessing catastrophizing, which other researchers have shown to be correlated with kinesiophobia, pain and functional limitations. If it was included, the analysis would be even more complete.

A more general limitation is the lack of cut-off points directly dedicated to TSK-TMD in the available literature. Due to this, the authors tried to adapt the scale with cut-off points from TSK-17 despite the strategy being, at the time of writing, unsupported and in need of future validation.

## 5. Conclusions

This paper’s findings displayed significantly higher levels of kinesiophobia, emotional state (except anxiety), functional state, and chronic pain in subjects with temporomandibular disorders, when compared to asymptomatic controls. A correlation between fear of movement and functional limitations of jaw function, mastication, mobility, and communication was also shown.

All TMD and nTMD subjects included in the study showed moderate (38.8% and 93.6, respectively) and high risk (61.7% and 6.4% respectively) of kinesiophobia, and so the prevalence was established at 100%; however, due to various factors, these results cannot be generalized to the whole TMD population.

Further studies should address three of the main weaknesses of this study; first, the study sample should be larger; second, the assessment of catastrophizing levels should complement kinesiophobia characteristics in the TMD population; third, cut-off points dedicated to the TSK-TMD questionnaire should be developed. The creation of subscales that determine levels of kinesiophobia would help the risk assessment to classify patients, and ensure their treatment plans are better addressed.

## Figures and Tables

**Table 1:** Kinesiophobia prevalence in the TMD and nTMD groups.

TSK-TMD		TMD Group			nTMD Group		
Level of Kinesiophobia	Points	n = 47			n = 47		
		n	%	Mean ± SD	n	%	Mean ± SD
low risk	<17	-	-	-	-	-	-
moderate risk	17–37	18	38.3	27.72 ± 6.37	44	93.6	21.50 ± 4.11
high risk	>37	29	61.7	44.69 ± 6.27	3	6.4	47.00 ± 6.08

Legend: TMD group—patients with temporomandibular disorders; nTMD group—patients with no signs of temporomandibular disorders; TSK-TMD—Tampa scale for kinesiophobia for temporomandibular disorders.

**Table 2:** Kinesiophobia, emotional state, functional state, and chronic pain differences between the TMD and nTMD groups.

Questionnaire	TMD Group			nTMD Group					
	n = 47			n = 47					
	Mean ± SD	Me	CI 95%	Mean ± SD	Me	CI 95%	Z	*p*	R
TSK-TMD	38.19 ± 10.41	39.0	35.13–41.25	23.12 ± 7.56	21.0	20.91–25.35	6.03	<0.001	0.723
PHQ-9	7.38 ± 4.64	7.0	6.02–8.75	3.94 ± 3.74	3.0	2.84–5.04	3.78	<0.001	0.453
GAD-7	5.81 ± 4.87	5.0	4.38–7.24	4.15 ± 4.68	3.0	2.77–5.52	1.96	0.050	0.235
JFLS-20	1.08 ± 1.04	0.83	0.78–1.39	0.04 ± 0.15	0.0	−0.01–0.08	6.16	<0.001	0.737
GCPS (CPI)	24.89 ± 21.21	30	18.67–31.12	1.84 ± 7.35	0.0	−0.31–4.00	5.13	<0.001	0.615

Legend: TMD group—patients with temporomandibular disorders; nTMD group—patients with no signs of temporomandibular disorders; TSK-TMD—Tampa scale for kinesiophobia for temporomandibular disorders; PHQ-9—patient health questionnaire; GAD-7—generalized anxiety disorder; JFLS-20—jaw functional limitation scale; GCPS (CPI)—GCPS graded chronic pain scale, CPI—characteristic pain Intensity score; CI—confidence interval; Me—median.

**Table 3:** Correlation coefficient values for kinesiophobia (TSK-TMD) levels.

	PHQ-9	GAD-7	JFLS-20	GCPS (CPI)
TSK-TMD	r = −0.07;	r = 0.022;	r = 0.621;	r = 0.101;
	*p* = 0.639	*p* = 0.886	*p* < 0.001	*p* = 0.501

Legend: TSK-TMD—Tampa scale for kinesiophobia for temporomandibular disorders; PHQ-9—patient health questionnaire; GAD-7—generalized anxiety disorder; JFLS-20—jaw functional limitation scale; GCPS (CPI)—GCPS-graded chronic pain scale, CPI—characteristic pain intensity score.

## Data Availability

Due to privacy restrictions, data is available on request from the corresponding author.
